# Bridging the antigen-presentation gap for adoptive cell therapies

**DOI:** 10.1016/j.omton.2024.200877

**Published:** 2024-09-26

**Authors:** Amy B. Hont, C. Russell Y. Cruz

**Affiliations:** 1 Center for Cancer and Immunology Research, Children’s National Hospital, Washington, DC, USA

## Main text

Adoptive cell therapy using polyclonal, antigen-specific T cells has revolutionized the treatment of infections, particularly against adenovirus, Epstein-Barr virus, and cytomegalovirus (CMV) post-hematopoietic stem cell transplantation.^1^ This therapy has led to remarkable outcomes, including the elimination of high-risk viruses and the cure of refractory virus-associated malignancies. However, recent efforts to expand the applicability of adoptive cell therapy to other diseases have stalled. While generating chimeric antigen receptor T (CAR-T) cells and virus-specific T cells (VSTs) has become more efficient, creating antigen-specific T cell products for low-frequency antigens or multiple antigens remains challenging. This process is often time-consuming and can lead to T cell exhaustion, limiting the therapeutic potential of adoptive cell therapy.^2^

In this issue of *Molecular Therapy Oncology*, Wu et al. propose a novel antigen-presenting cell (APC)-mimic fusion protein to overcome these limitations.^3^ The APC-mimic fusion protein comprises the cognate peptide-human leukocyte antigen complex and the co-stimulatory CD80 protein, each in two halves of a chimeric immunoglobulin G1 protein. APC-mimic can efficiently expand antigen-specific T cells both *in vitro* and *in vivo*. This has the potential to enhance cell therapy and immunotherapy approaches for various diseases.

While *ex vivo* expansion can lead to T cell exhaustion, the authors show that APC-mimic may promote a more functional T cell response. They show T cell activation in response to the chimeric protein, expansion of antigen-specific populations in culture, and functional activity of APC-mimic T cells *in vitro* (using CMV pp65 as a model antigen). Furthermore, the authors show that the activation influences the differentiation process of T cells and can amplify specific clonotypes. The ability of the APC-mimic to generate antigen-specific T cells *in vivo* without peripheral blood collection is particularly promising. Using the model OT-1 antigen, the artificial APC has shown efficacy in controlling tumors without T cell exhaustion in preclinical models.^3^

The described approach with APC-mimic offers several advantages as an antigen-presentation system. As an antigen-agnostic approach, it can be used to present a wide range of antigens and is thus easily adapted to target different diseases. As a non-cellular system, it offers the benefit of decreased biological variability, translating to great consistency and predictability in manufacturing processes. This system can also be easier to store and transport. Additionally, the potential for direct administration *in vivo* allows it to bypass the need for *ex vivo* manipulation and the complexities involved with this process.

There are limitations to this proposed technology. The idea and design have been investigated and may be difficult to differentiate from existing artificial APCs on the market. The authors also demonstrate the potential development of exhaustion with *ex vivo* application of APC-mimic, with increased expression of markers associated with terminally exhausted T cells (e.g., PD1, LAG-3), although the same cell population exhibits increased cytotoxicity to pp65^+^ targets *in vitro*, supporting efficacy. They also describe increased populations of effector memory T (Tem) cells with exhausted T (Tex) cells, consistent with the expected phenotype of antigen-specific T cells expanded *ex vivo*. The functional implications of this phenotype are not entirely clear, but they raise the question of optimizing this process to minimize exhaustion (e.g., new co-stimulatory ligands) or combine therapy with immune checkpoint inhibition.^3^

APC-mimic has thus far been tested exclusively in systems that are based on memory T cells without significant manufacturing hurdles. Both CMV pp65 and OT-1-specific T cells are typically readily expandable cell products, and the strong antigens in each may be responsible for shifting the population to a more homogeneous/monoclonal composition. Additionally, the use of a peptide-major histocompatibility complex rather than a pepmix of overlapping peptides, which is more likely to generate a diverse, polyclonal T cell population, would support this monoclonal response. This may be beneficial if supportive of a potent antigen-specific response, but in the context of tumor antigens, it may correspond to an increased risk of immune escape. The authors note that this approach is readily translatable and thus may lend itself to the relatively simple generation of multiple APC-mimic to use simultaneously, thereby generating a more diverse antigen-specific response to address this challenge.

The lack of mechanotransduction, a critical process in T cell stimulation, particularly with less-potent antigens, could also hinder its efficacy. Recent findings have emphasized the role of immune receptors as essential mechanotransducers, converting mechanical forces due to membrane attachment and binding into biochemical signals that are vital steps in T cell activation, as they immobilize signal 1 and 2 components on nanoparticles that mimic the cell surface.^4^ Indeed, the topology of cells on these nanoplatforms has been shown to influence T cell activation.^5^

While it has not been directly compared to other strategies, a particularly enticing application of APC-mimic is its application *in vivo* to generate an antigen-specific response without the requirement of peripheral blood collection/apheresis and *ex vivo* expansion. When the APC-mimic strategy was applied in the context of pp65^+^ K562 tumors, the authors describe excellent tumor control with the retention of Tem cells in the spleen, tumors, and peripheral blood collected, with a corresponding decrease in the frequency of PD1^+^ and TIM3^+^ cells. This suggests the enhancement of a functional T cell response *in vivo* without exhaustion or anergy. Further investigation of the utility of this approach in cancer treatment found that combining APC-mimic infusion with ovalbumin (OVA)-specific T cells was able to control tumor growth compared to OVA-specific T cells alone in a murine model (OT-1). This holds great promise in the incorporation of APC-mimic to current immunotherapeutic strategies, particularly adoptive cell therapy, the progress of which has stalled in treating refractory solid tumors. A similar study has also demonstrated that artificial APCs (aAPCs) can effectively boost the *in vivo* efficacy of low-avidity tumor-specific cytotoxic T lymphocytes (CTLs) in mouse models of lung melanoma.^6^ In this context, aAPC treatment significantly reduced tumor growth and even achieved complete eradication in certain cases when combined with the adoptive transfer of low-avidity CTLs.

To overcome these limitations, further research and development are essential to ensure the effectiveness and viability of the technology in real-world applications. Nevertheless, APC-mimic is a promising mechanism to enhance the antigen-specific T cell response. Ultimately, this approach may play a crucial role in advancing targeted therapies for high-risk diseases with the potential to expand effective therapeutic options for high-risk patients.

## Acknowledgments

A.B.H. has received support from the Department of Defense Peer-Reviewed Cancer Research Program (W81XWH2110259). C.R.Y.C. and A.B.H. have received support from the Cancer Grand Challenges grant awarded by Cancer Research United Kingdom and the National Cancer Institute of the National Institutes of Health (1OT2CA278200-01).

## Author contributions

A.B.H. and C.R.Y.C. conceived and wrote this commentary.

## Declaration of interests

C.R.Y.C. is a co-founder and Scientific Advisory Board member of Mana Therapeutics.
